# Predictive value of hematological markers of systemic inflammation for managing cervical cancer

**DOI:** 10.18632/oncotarget.14827

**Published:** 2017-01-26

**Authors:** Lin Wang, Jing Jia, Lu Lin, Junying Guo, Xingming Ye, Xiongwei Zheng, Ying Chen

**Affiliations:** ^1^ Fujian Provincial Cancer Hospital, The Affiliated Cancer Hospital of Fujian Medical University, Fuzhou, China

**Keywords:** Cervical cancer, metastasis, neutrophil/lymphocyte ratio, platelet/lymphocyte ratio

## Abstract

The neutrophil/lymphocyte ratio (NLR), platelet/lymphocyte ratio (PLR), and red cell distribution width (RDW) are markers of systemic inflammation with prognostic significance for cancers. The aim of the study was to investigate the predictive significance of pretreatment values of NLR, PLR, and RDW in cervical cancer. We retrospectively analyzed 515 patients with cancer. Median values of NLR and PLR were higher in patients with cancer compared with controls and were consistently elevated during tumor progression, while the RDW was uninformative. Increased NLR was associated with lymph node (LN) metastasis and depth of stromal infiltration, and increased PLR correlated only with LN metastasis. The pretreatment NLR or PLR value was a significant predictor of LN metastasis, which enhanced when NLR and PLR values were combined. Further, NLR and PLR were as effective as squamous cell carcinoma antigen (SCC-Ag) for predicting distant tumor metastasis. However, no prognostic significance of NLR or PLR was found in the patients with early cancer stages. Our study suggested that pretreatment values of NLR and PLR might be helpful to predict the presence of distant and LN metastasis in patients with cervical carcinoma, but not adequate prognostic factors for early stage patients.

## INTRODUCTION

Cervical cancer is the third most common malignancy of women worldwide, and approximately 250,000 patients die from this disease each year [1, 2]. The incidence and mortality rate of cervical cancer are much higher in less developed countries, in part, because of poor sanitation, insufficient medical infrastructure, and economic conditions [2].

Inflammation is a key characteristic of the tumor microenvironment and plays a central role in the initiation, promotion, progression, invasion, and metastasis of a tumor [3]. The prognostic and predictive values of markers of systemic inflammation such as serum C-reactive protein (CRP), which is included in the Glasgow Prognostic Score (GPS), are established for numerous malignant tumors [4, 5]. Recently, hematological markers of inflammation that are readily measured in the clinic, such as the neutrophil/lymphocyte ratio (NLR), platelet/lymphocyte ratio (PLR), and red cell distribution width (RDW) are attracting increased interest because of their predictive or prognostic value for evaluating numerous cancers, e.g. non-small cell lung cancer, pancreatic adenocarcinoma, gastric cancer, and renal cell carcinoma [6–11]. However, their utility for managing cervical cancer remains to be determined.

Human papillomavirus infection is the most important causative factor in cervical carcinogenesis, and inflammatory pathways play an important role in tumorigenesis and progression [12]. Three published studies evaluated the predictive significance of NLR and PLR for cervical cancer. The limited data indicated that elevated pretreatment values of NLR and PLR correlate with unfavorable histopathological characteristics [13] and poor prognosis for survival or response to radiation therapy [13, 14]. However, not all investigators reached the same conclusion [15]. Because of the limitation of previous sample sizes, a comprehensive analysis of the association of these markers with cancer clinicopathological features would help establish their potential for managing cervical cancer.

Therefore, the aim of the present study was to investigate the predictive values of NLR, PLR, and RDW in patients with cervical cancer. We assessed correlations between pretreatment levels of these markers with tumor stage and clinicopathological characteristics of mainly squamous cell cervical carcinoma, which accounts for the majority of cervical cancers. A parallel analysis of the serum levels of squamous cell carcinoma antigen (SCC-Ag) was conducted as a reference.

## RESULTS

### Patient characteristics

We enrolled 515 patients with cervical squamous cell carcinoma. Their median age was 51 years (range, 25-79 years) and 119 (23.1%), 342 (66.4%), 44 (8.5%), and 10 (1.9%) patients were diagnosed with FIGO stages I-IV, respectively. Serum SCC-Ag levels were available for 501 patients (97.3%), and 425 (82.5%) and 90 (17.5%) had tumors with histological grade G1/G2 or G3, respectively.

### Comparison of laboratory parameters of patients with cervical cancer with those of healthy controls

The laboratory parameters of patients with cancer and healthy controls are shown in Table 1. Cancer patients had significantly higher serum SCC-Ag levels and total white blood cell (WBC), neutrophil, and platelet counts. The difference in lymphocyte counts was smaller but significant. Hemoglobin (Hb) levels were significantly lower in patients with cancer. The median values of NLR and PLR were higher in patients with cancer, but there was no difference in the RDW between groups. Analysis of receiver operating characteristic (ROC) curves yielded the cutoff values of 2.27 for NLR (area under the curve [AUC], 0.570; sensitivity, 38.64%; specificity, 82.48%), and 148.9 for PLR (AUC, 0.566; sensitivity, 32.04%; specificity, 88.54%), which were used to discriminate patients with cancer from controls. The relative SCC-Ag cutoff value was 1.5 (AUC, 0.900; sensitivity, 66.87%; specificity, 96.27%).

**Table 1:** Comparison of laboratory parameters in cervical cancer patients with healthy controls

	controls	cervical cancer patients	*P* value
	314	515	
FIGO stage (I/II/III/IV)		119/342/44/10	
WBC (10^9^/L)	5.8 (5.1-6.6)	6.8 (5.6-8.3)	**<0.0001**
Neutrophil (10^9^/L)	3.38 (2.94-4.01)	3.97 (3.11-5.23)	**<0.0001**
Lymphocyte (10^9^/L)	1.89 (1.66-2.18)	1.94 (1.63-2.39)	**0.0367**
Platelet (10^9^/L)	225 (198-251.3)	247 (208-297)	**<0.0001**
Hb (g/L)	128 (123-134)	124 (114-131)	**<0.0001**
RDW (%)	12.9 (12.6-13.3)	12.9 (12.4-13.6)	0.8667
NLR	1.80 (1.49-2.16)	1.93 (1.48-2.70)	**0.0007**
PLR	115.4 (100.7-137.1)	125.9 (97.62-157.8)	**0.0013**
SCC-Ag (ug/L)	0.7 (0.5-0.9)	2.8 (1.2-7.4)	**<0.0001**

### Correlation between laboratory parameters and clinicopathological characteristics of patients with cervical cancer

Patients were stratified according to the cutoff values obtained using ROC analysis, and the significance of the associations of clinicopathological characteristics with pretreatment NLR, PLR, and SCC-Ag were determined using Fisher's exact test (Table 2). Histologic grades did not correlate with NLR or PLR. NLRs > 2.27 were associated with lymph node (LN) metastasis (*P* < 0.0001) and the depth of stromal infiltration (*P* = 0.029). PLRs > 148.9 positively correlated with LN metastasis (*P* = 0.016). NLR or PLR values did not correlate significantly with tumor size. In contrast, SCC-Ag values > 1.5 positively correlated with increased tumor size, depth of stromal infiltration, and LN metastasis (all *P* < 0.0001).

**Table 2:** The relationship of NLR, PLR, and SCC-Ag with clinicopathological characteristics in cervical cancer patients

Variables	No. Patients	NLR		*P* value	PLR		*P* value	SCC-Ag		*P* value
		>=2.27	<2.27		>=148.9	<148.9		>=1.5	<1.5	
Histologic grade										
G1/G2	425	165	260	0.9054	132	293	0.3202	296	119	0.3648
G3	90	34	56		33	57		57	29	
Tumor size (cm)										
<4	99	30	69	0.0662	26	73	0.1887	24	67	**< 0.0001**
>4	416	169	247		139	277		329	81	
Depth of stromal infiltration										
<1/2	171	52	119	**0.0296**	48	123	0.3079	75	89	**< 0.0001**
>1/2	193	80	113		64	129		158	26	
unknown	151	67	84		53	98		120	29	
LN metastasis										
NO	271	78	193	**< 0.0001**	74	197	**0.0171**	142	120	**< 0.0001**
Yes	117	60	57		47	70		102	12	
unknown	127	61	66		44	83				

**Figure 1:**
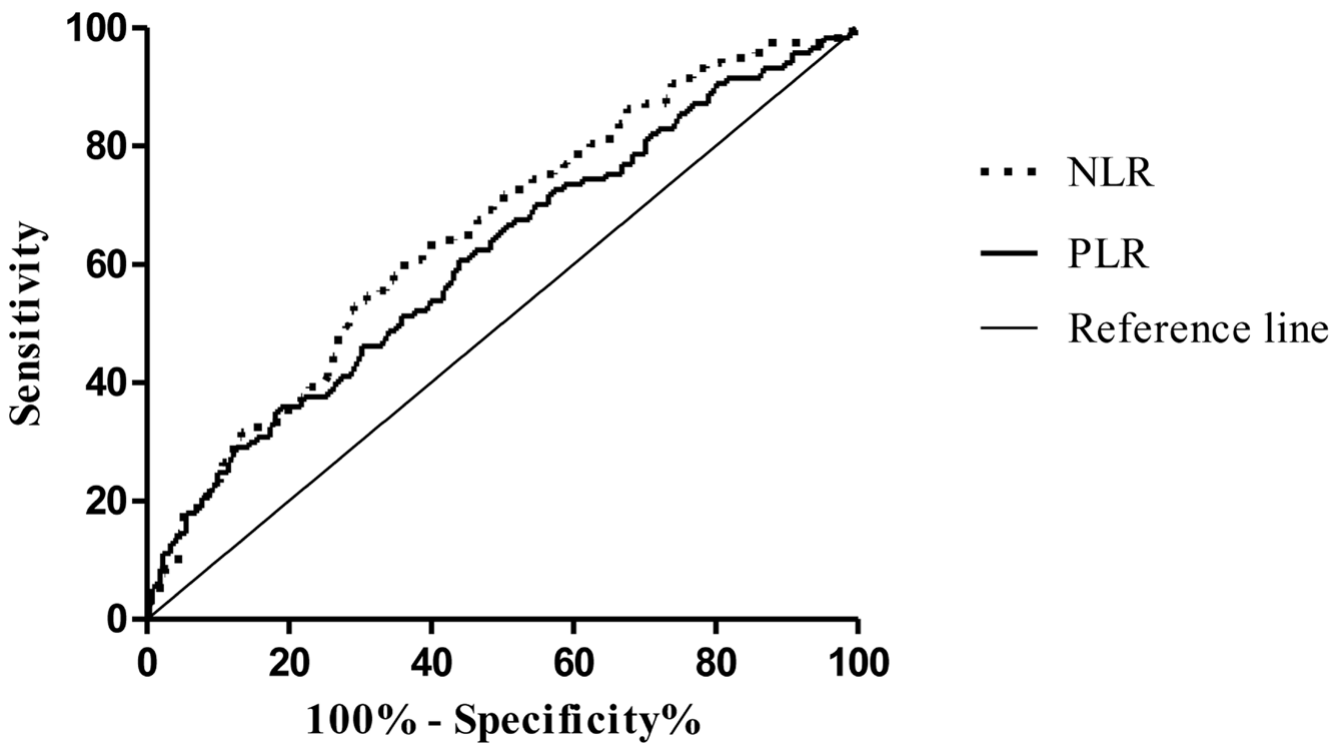
ROC curves of pretreatment NLR and PLR for patients with or without LN metastasis

ROC analysis of the ability of NLR and PLR to predict LN metastasis (Figure 1) yielded AUC values of 0.652 and 0.614, respectively. The odds ratios (ORs) for the prediction of LN metastasis using NLR, PLR, and NLR + PLR are shown in Table 3.

**Table 3:** Odds ratios for the exposure of LN metastasis determined by the NLR or PLR

Parameter	OR	95 % CI	*P* value
NLR	2.759	1.766 to 4.310	< 0.0001
PLR	2.327	1.430 to 3.787	0.0006
NLR+PLR	2.836	1.687 to 4.766	< 0.0001

### Association of laboratory parameters with FIGO stages of patients with cervical cancer

**Figure 2:**
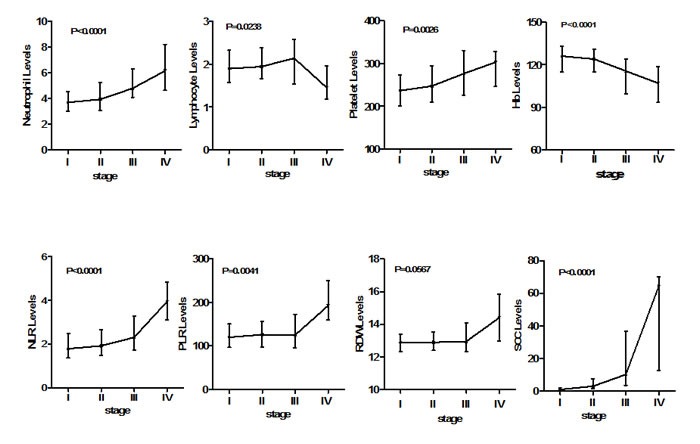
Association of laboratory parameters with FIGO stages in cervical cancer patients

Kruskal-Wallis analysis (Figure 2) revealed that increases in NLR (*P* < 0.0001), PLR (*P* = 0.0041), and SCC-Ag (*P* < 0.0001) were consistently and significantly associated with advanced cervical cancer stage as well as with neutrophil and platelet counts. The median differences in neutrophil counts compared with those of controls were 9.4% (Stage I), 16.4% (Stage II), 41.7% (Stage III), and 82% (Stage IV). The corresponding differences in platelet counts were 5%, 10%, 22.9%, and 34.7%. However, the lymphocyte count declined sharply by stage IV after a steady increase from stages I to III. The differences in median lymphocyte count compared with that of controls were 0.04%, 3.0%, 13.5% and −22.8%. In contrast, Hb levels decreased continuously with advanced tumor stage. No significant interaction between RDW and tumor stage was detected.

The therapeutic options for cervical cancer patients vary according to FIGO stage. Patients with tumor stage below IIB are advised to undergo surgery, and patients with stage IIB and above are to be treated with adjuvant radiochemotherapy. We used ROC analysis to determine the predictive significance of pretreatment values of NLR, PLR, RDW, and SCC-Ag for tumor staging. As shown in Table 4, the predictive efficacy of SCC-Ag was best for all tumor stages among the markers studied. Although NLR and PLR were moderately effective for patients with stages IIB and III+IV, no predictive power was found for patients with stages earlier than IIB. In addition, RDW was uninformative.

**Table 4:** Area under the ROC analysis curve of pretreatment PLR, NLR, RDW and SCC-Ag for FIGO stage in cervical cancer patients

	NLR		PLR		RDW		SCC-Ag	
Stage	AUC	*P* value	AUC	*P* value	AUC	*P* value	AUC	*P* value
Stage I+IIA	0.5153	0.5112	0.5360	0.1232	0.5150	0.5205	0.8568	< 0.0001
Stage IIB	0.6144	< 0.0001	0.5993	0.0004	0.5037	0.8937	0.9554	< 0.0001
Stage III+IV	0.7423	< 0.0001	0.6381	0.0012	0.5612	0.1510	0.9673	< 0.0001

### Values of NLR and PLR for predicting the patient survival

An independent group of 129 cancer patients who underwent surgical treatment was selected, for determining the prognosis value of preoperative NLR or PLR in early stage cancer patients. The median follow-up was 64 months (range, 6-142 months). 11 of 129 patients (8.5%) were died during the follow-up period, and the overall survival rate was 91.5%. Using the cutoff values of 2.27 for NLR and 148.9 for PLR, the Kaplan-Meier survival curves in Figure 4 indicated that NLR (*P* = 0.7124; HR, 0.7968; 95% CI of ratio, 0.2383-2.665) and PLR (*P* = 0.1674; HR, 0.4169; 95% CI of ratio, 0.1204-1.444) exhibited no significant difference on overall survival (OS), which are not adequate prognostic factors for predicting the OS of patients with early stage cervical cancer.

**Figure 3:**
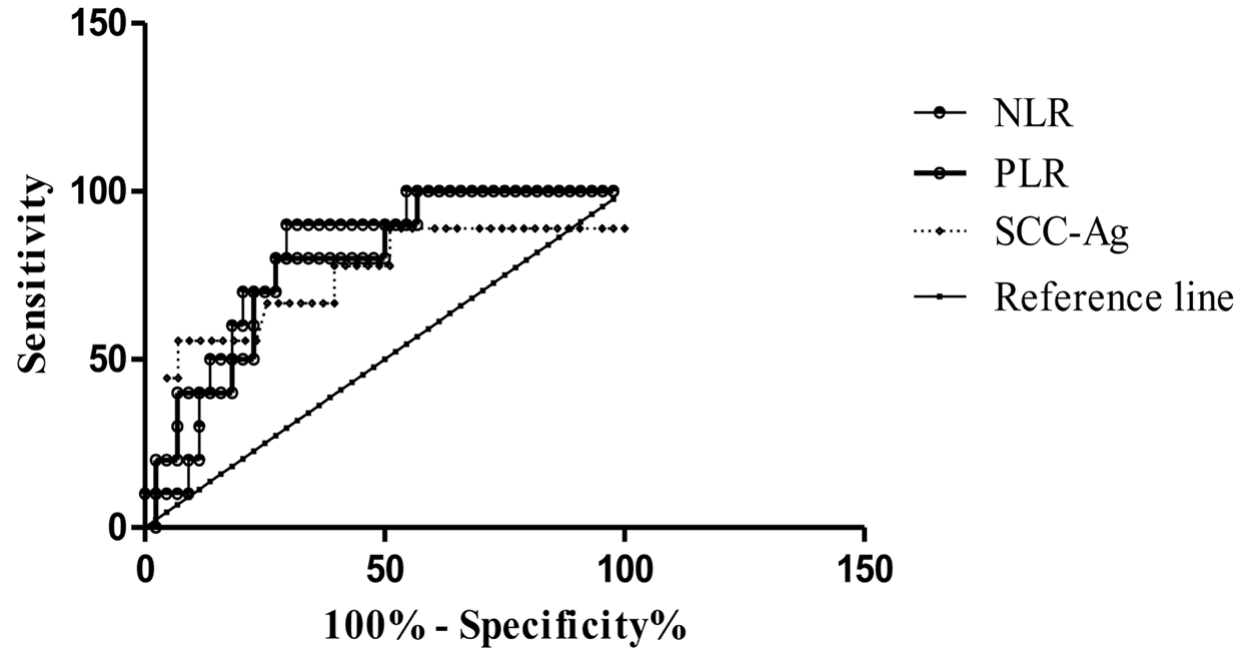
ROC curves of pretreatment NLR, PLR, and SCC-Ag in cancer patients with FIGO stage III and stage IV

**Figure 4:**
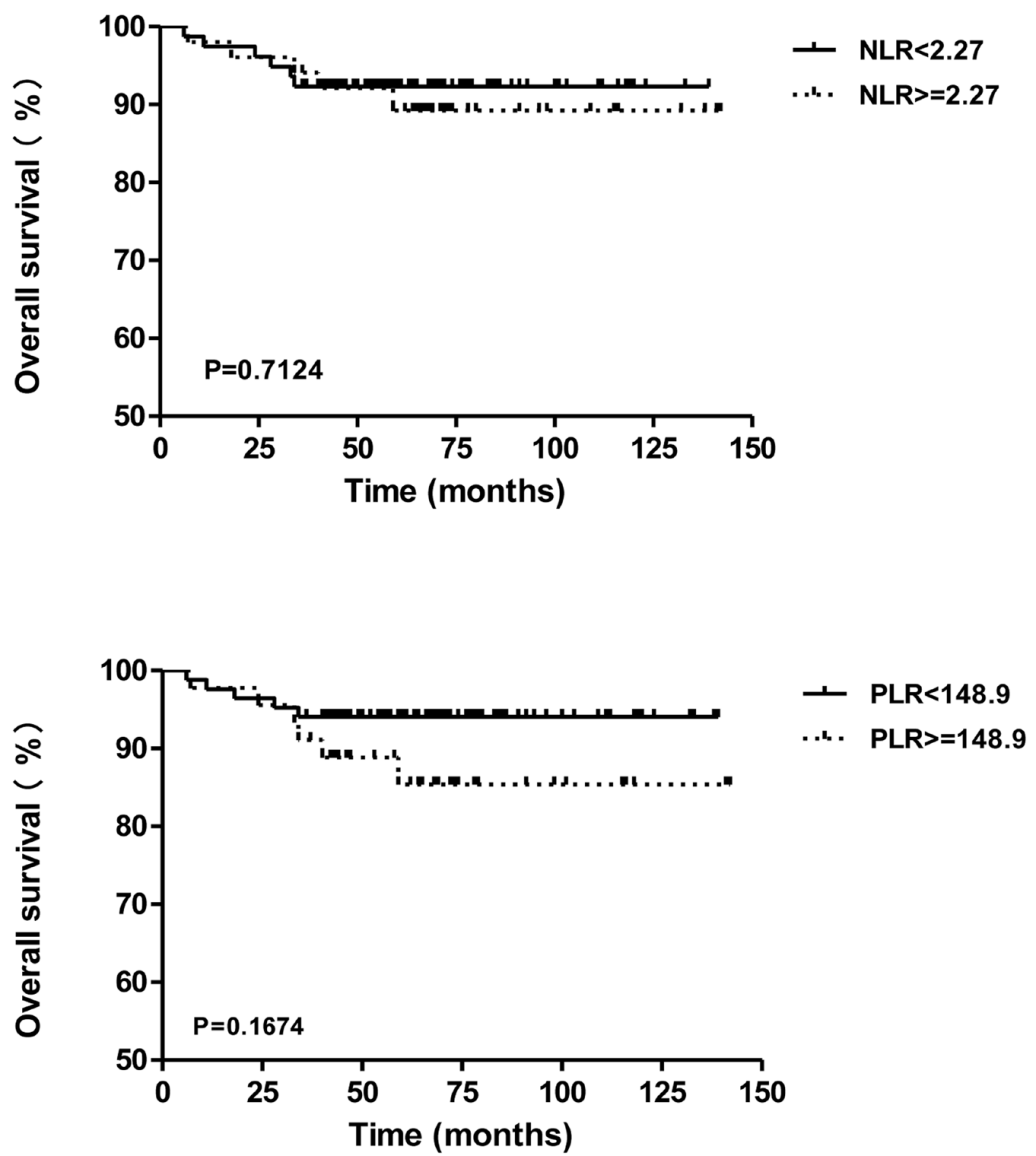
Overall survival of cervical cancer patients in different subgroups of NLR or PLR

### Significance of NLR and PLR for predicting distant metastasis

The data revealed a dramatic decline in lymphocyte counts and a significant increase in neutrophil and platelet counts in patients with stage IV disease. Therefore, we used ROC analysis to assess the significance of NLR and PLR for predicting distant metastasis. The data indicated that NLR (AUC, 0.804; sensitivity, 90%; specificity, 70.45%) and PLR (AUC, 0.784; sensitivity, 80%; specificity, 72.73%) effectively discriminated patients with stage III from those with stage IV disease (Figure 3). These results were comparable to or exceeded the predictive significance of SCC-Ag (AUC, 0.743; sensitivity, 55.56%; specificity, 93.02%). The relative cutoff values were 2.99 and 168.7 for NLR and PLR, respectively.

## DISCUSSION

Inflammation plays a key role in tumorigenesis and in the progression of cervical cancer [12]. In the present study, we focused on determining the predictive significance of the markers of inflammation NLR, PLR, and RDW for squamous cell cervical carcinoma. We found that pretreatment values of NLR and PLR were significantly higher in patients with cancer compared with controls, and they increased steadily with advanced cancer stage. The present data indicate the difference in inflammation status between patients with cervical cancer compared with healthy subjects and suggest a continuously progressive inflammatory response in patients as disease advances. Moreover, we discovered that NLR and PLR were significant predictors of either LN or distant metastasis. However, RDW was uninformative.

In patients with cervical cancer, high pretreatment NLR is associated with poor prognosis of survival [13, 14], which may be explained by our observation here that a higher NLR was associated with more advanced stages of cancer that are typically associated with poor prognosis. The association of NLR and PLR with cervical cancer stage is consistent with our previous findings in a study of patients with colorectal cancer [16], which assessed the relationship of markers of inflammation with clinicopathological characteristics. The associations of NLR with the depth of stromal infiltration, histologic grade, and tumor size agree with the findings of a previous report [13], except that we did not detect an association of PLR with tumor size [13]. This discrepancy might be attributed to differences in patients’ characteristics or study designs.

In the present study, we found that NLR and PLR had significant power to predict patients with tumor stages IIB and above as well as LN metastasis. Such patients typically choose to undergo adjuvant treatment *vs*. surgery. LN metastasis is an independent prognostic factor for patients with cancer [17]. Thus, our results highlight the clinical value of both markers for therapeutic decision-making or determining prognosis. Moreover, both markers showed promising efficacy for predicting distant metastasis (stage IV), which was comparable to or exceeded that of SCC-Ag.

The mechanisms underlying our observations are unclear. Changes in NLR and PLR reflect the balance between host neutrophil- and platelet-dependent inflammatory responses and lymphocyte-mediated antitumor immune responses. Neutrophils and platelets participate in tumor progression and metastasis [18–22], and circulating lymphocytes play an important role in preventing the proliferation and metastasis of tumor cells [23]. In the present study, the high NLR and PLR values associated with tumor stages were associated with steady increases of neutrophil and platelet counts compared with those of lymphocytes in all tumor stages. These results reflect ongoing dysfunction of the patient's immune system. A significant finding of our study is that lymphocyte counts dramatically decreased in patients with stage IV disease, suggesting dysfunction of the host's antitumor immune response. This may explain the increasing ability of NLR and PLR to predict the presence of distant metastasis. We hypothesize therefore that a collapse of host immune surveillance was partly responsible for the development of distant metastasis in our patients with cervical cancer.

SCC-Ag serves as a marker for cervical cancer as well as a predictive and prognostic factor for squamous cell cervical carcinoma [24]. Pretreatment SCC-Ag levels correlate with FIGO stage [25–27] and pelvic LN metastasis [28]. In the present study, the predictive significance of SCC-Ag was consistent with a that of a previous study [29] and exceeded that of NLR and PLR, except for predicting distant metastasis. However, laboratory testing for SCC-Ag requires special equipment such as an electrochemiluminescence immunoassay system, which might be costly and less implemented. In contrast, NLR and PLR can be routinely determined from blood counts, which are widely available. Therefore, NLR and PLR will likely serve as alternative or supplementary markers in clinical practice.

RDW is traditionally used for the differential diagnosis of microcytic anemia and was recently proposed as a marker of systemic inflammation for numerous chronic inflammatory diseases [30, 31]. Further, patients with breast, renal, or colon cancer have higher RDW values compared with healthy controls [32–34]. Moreover, elevation of RDW is associated with cancer stage, histopathological characteristics, or worse prognosis in patients with certain solid tumors, e.g. lung cancer [35]. In the present study, we did not detect a significant difference in RDW levels between patients and controls and no association between RDW and tumor stages. Our data suggest therefore that the biological role of RDW in cervical cancer may differ for other cancers. We note that severe anemia was not present in our patients. For example, in patients with stage IV disease, the median level of hemoglobin was 107 g/L, which might explain the unchanged RDW values. We conclude therefore that RDW was not suitable for predicting the presence or stage of cervical cancer.

Although the present study indicates the potential value of NLR and PLR in clinical practice, it has several limitations. First, the study was retrospective. Second, there were few patients with advanced disease, which might have introduced bias in judging the value of these markers, particularly the value of both markers for predicting distant metastasis. Thus, our observations must be validated by a large-scale prospective study. Moreover, it remains to be determined whether NLR or PLR serve as a predictive marker for therapeutic outcomes.

Our preliminary analysis of patients who underwent surgery (Stage I + IIA) who had pretreatment values of NLR or PLR over the cutoff, reveals a significant decline in the median value of NLR after surgery (Supplementary Data A and B), although the values are inconsistent among individuals. For example, 25% (17/68) exhibited a further increase in NLR after surgery, although the value decreased in the 75% (51/68) of patients. Determining the clinical significance of the difference will be an interesting topic for a future study.

Of noted, the prognostic values of NLR or PLR have been observed in various types of cancers [7–10]. In cervical cancer, Lee et al found that the pretreatment NLR is an independent survival factor [11]. However, in another two studies which includes patients with stage I - II [13] and IB2 - IIB [15], no significant efficacy of NLR or PLR was found for predicting the OS. This disagreement might result from the different study populations among them, since the former study consisted with patients at stages from IB to IVA. Different therapeutic choices associated with the patient conditions could attribute to diversity of the study results. To test the hypothesis, the present study analyzed the prognostic value of NLR and PLR in an independent patient group with early stage of cervical cancer (stages IB to IIA), which has been followed up for more than five years. Our data showed that both NLR and PLR are not adequate prognostic factors for predicting the OS in patients at early stage, which were consistent with those of previous studies [13, 15]. Those finding might be explained by the relative less elevation of NLR and PLR in early stage patients, as we have shown in Table 4. In addition, since various therapies were chosen in the relapsed conditions in the studies included here, there may be no significant prognostic value according to NLR and PLR status in this meta-analysis.

In summary, we show here that pretreatment values of NLR and PLR might be helpful to predict the presence of distant and LN metastasis in patients with squamous cell cervical carcinoma, but not adequate prognostic factors for early stage patients.

## MATERIALS AND METHODS

### Study design

This retrospective study enrolled patients with cervical cancer who were admitted to the Fujian Provincial Cancer Hospital between January 2012 and May 2014. All patients were histologically diagnosed with squamous cell cervical carcinoma and staged according to International Federation of Gynecology and Obstetrics (FIGO) criteria. Patients were excluded if they were previously treated for cervical cancer or had concurrent hematologic, autoimmune, or infectious disease. Patient characteristics and pretreatment hematological parameters, serum SCC-Ag levels, and available pathological features were collected from the each patient's medical records. A group of 314 age-matched healthy women who visited the hospital for routine evaluations served as controls. In addition, an independent group of 129 cervical cancer patients at stages IB (30 cases) to IIA (99 cases) was enrolled for the survival analysis. All of them were underwent surgical treatment during the period of 2005 to 2010 at our hospital. The Institutional Ethics Committee approved this study.

### Calculation of NLR and PLR

NLR was defined as the absolute number (10^3^ cells/mL) of neutrophils divided by the absolute number of lymphocytes, and PLR was calculated by dividing the absolute number of platelets by the absolute number of lymphocytes.

### Statistical analysis

Data are expressed as the median ± interquartile range. GraphPad Prism 5 (http://www.graphpad.com/scientific-software/prism/) and SPSS version 17.0 (SSPS Inc., Chicago, IL, USA) were used for statistical analysis. The significance of the differences in laboratory parameters between patients and controls was evaluated using the Mann-Whitney U test. The associations of NLR, PLR, and SCC-Ag with clinicopathologic variables were analyzed using the Fisher's exact test, and the associations of laboratory parameters with tumor stages were assessed using the Kruskal-Wallis test. Receiver operating characteristic curve (ROC) analysis was used to evaluate the predictive significance of NLR, PLR, and SCC-Ag to discriminate between patients with cancer from healthy subjects, patients with different tumor stages, and patients with or without LN metastasis and distant metastasis. The overall survival (OS) curve was estimated by Kaplan-Meier analysis and the log-rank test was carried out to evaluate the statistical significance in survival between different subgroups. Statistical significance was defined as *P* < 0.05 (two-tailed).

## SUPPLEMENTARY MATERIALS FIGURE AND TABLE



## Abbreviations

NLR: neutrophil-lymphocyte ratio
PLR: platelet-lymphocyte ratio
RDW: red cell distribution width
SCC-Ag: squamous cell carcinoma antigen
FIGO: International Federation of Gynecology and Obstetrics
AUC: area under curve
ROC: receiver operating characteristic curve
WBC: absolute white blood cell
LN: lymph node
OS: overall survival
