# Supplementary material for: Predictive value of hematological markers of systemic inflammation for managing cervical cancer
Source: Oncotarget. 2017 Jan 26;8(27):44824–32. doi: 10.18632/oncotarget.14827 (PMC5546522; doi:10.18632/oncotarget.14827)
Supplement: Supplementary file 1 [file oncotarget-08-44824-s001.pdf]

Predictive value of hematological markers of systemic inflammation for managing cervical cancer

Supplemental data

A

Table. Changes of NLR and PLR after surgical treatment in patients with pretreatment levels of NLR>=2.27 or PLR>=148.9.

	pre-operation	*post-operation	*P* value
NLR (n=68)	2.92 (2.55-3.73)	2.30 (1.78-3.09)	<0.0001
PLR (n=75)	169 (155.5-194.4)	167.1 (121.9-238)	0.4126

There is a significant decrease in the median level of NLR after surgical operation, while not change in PLR. *Post-operation was defined by one month after surgery plus a complete course postoperative adjuvant treatment.

B

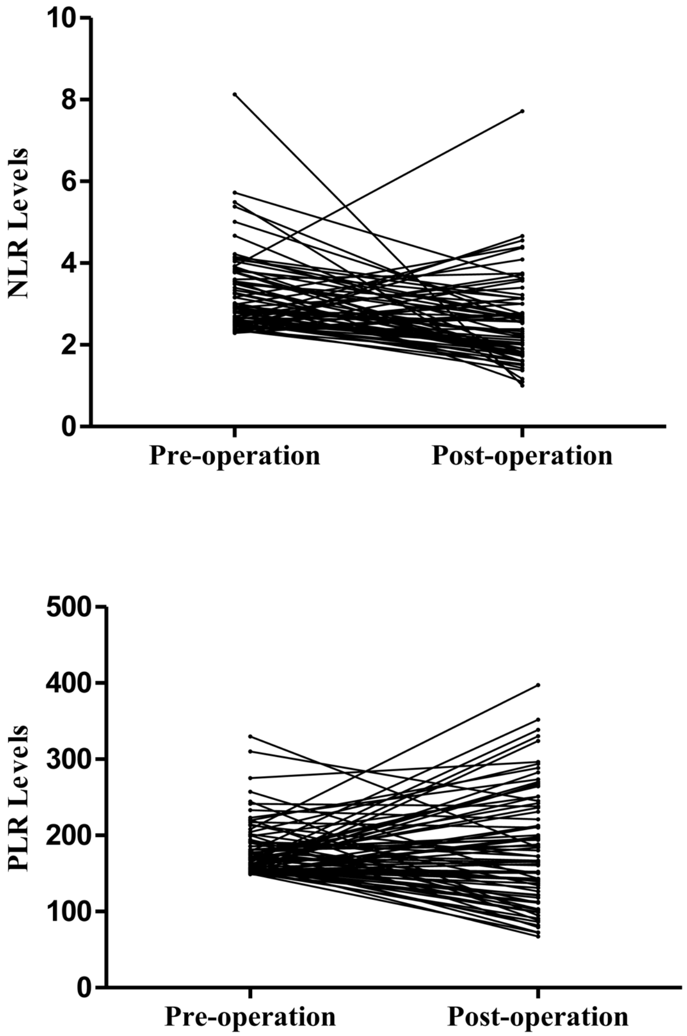

Figure. The changes of NLR and PLR before and after surgical operation.

Patients who had pretreatment levels of NLR>=2.27 (n=68) or PLR>=148.9 (n=75) were enrolled for the comparison. 17 of 68 patients showed an increase of NLR in post-operation, while the rest (51/68) manifested a decrease. The relative portion for PLR was 32 with increase and 43 with decrease.
